# Implementation and Performance Evaluation of Quantum-Inspired Clustering Scheme for Energy-Efficient WSNs

**DOI:** 10.3390/s25185872

**Published:** 2025-09-19

**Authors:** Chindiyababy Uthayakumar, Ramkumar Jayaraman, Hadi A. Raja, Kamran Daniel

**Affiliations:** 1 Department of Computing Technologies, SRM Institute of Science and Technology, Kattankulathur, Chennai 603203, India; ram.kumar537@gmail.com; 2 Department of Electrical Power Engineering and Mechatronics, Tallinn University of Technology, 19086 Tallinn, Estonia; kamran.daniel@taltech.ee

**Keywords:** clustering, wireless sensor network (WSN), QICS, quantum algorithm, grover algorithm, quantum grover

## Abstract

Advancements in communication technologies and the proliferation of smart devices have significantly increased the demand for wireless sensor networks (WSNs). These networks play an important role in the IoT environment. The wireless sensor network has many sensor nodes that are used to monitor the surrounding environment. Energy consumption is the main issue in WSN due to the difficulty in recharging or replacing batteries in the sensor nodes. Cluster head selection is one of the most effective approaches to reduce overall network energy consumption. In recent years, quantum technology has become a growing research area. Various quantum-based algorithms have been developed by researchers for clustering. This article introduces a novel, energy-efficient clustering scheme called the quantum-inspired clustering scheme (QICS), which is based on the Quantum Grover algorithm. It is mainly used to improve the performance of cluster head selection in a wireless sensor network. The research conducted simulations that compared the proposed cluster selection method against established algorithms, LEACH, GSACP, and EDS-KHO. The simulation environment used 100 nodes connected via specific energy and communication settings. QICS stands out as the superior clustering method since it extends the lifetime of the network by 30.5%, decreases energy usage by 22.4%, and increases the packet delivery ratios by 19.8%. The quantum method achieved an increase in speed with its clustering procedure. This study proves how quantum-inspired techniques have become an emerging approach to handling WSN energy restrictions, thus indicating future potential for IoT systems with energy awareness and scalability.

## 1. Introduction

**Figure 1:**
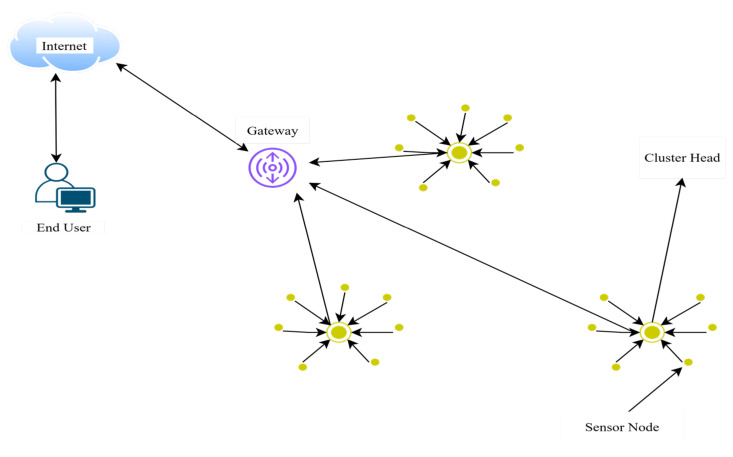
WSN clustering architecture.

A wireless sensor network (WSN) is a specialized collection of transducers that deliver sensory information while relying on limited battery resources. In recent times, there has been increased attention towards the issue of energy efficiency [1] and spectrum efficiency by operating in licensed and unlicensed bands [2,3]. This is primarily due to the challenges posed by the impracticality of frequently altering or recharging battery resources, particularly for networks operating on a large scale or within remote areas. Uneven distribution of sensor nodes often leads to the energy hole problem within the network [4]. One of the most effective ways to conserve energy is to keep placing nodes across the network area and cluster formation to ensure a balanced energy flow [5]. The cluster head gathers all detected data from the sensor nodes and subsequently performs the aggregation of the respective data. In Figure 1, a clustering architecture of WSNs is depicted.

In recent years, many distributed algorithms have been used to detect an uncovered area and redundant nodes on the network and randomly deploy the nodes in the proper manner [6]. The wireless sensor network is more energy-constrained and resource-limited. Clustering is one of the key features to manage energy consumption in WSN, which can divide the entire network into numerous distinct clusters [7]. Within each cluster, a designated cluster head (CH) assumes a central role. Basically, the designation of the cluster head can be achieved through random selection, taking into account the sensor population. The primary role of these sensors is to observe specific environmental parameters within their region and send these data to the cluster head, which collects the information to facilitate subsequent data aggregation procedures [8]. The utilization of data aggregation techniques serves the purpose of enhancing the network’s lifetime. The aggregated data, once consolidated, are forwarded to the base station (BS). The BS is normally powered up by an electrical grid, and it has more computational capabilities. The radio transmission rate can be reduced while simultaneously enhancing the network’s lifespan by using this clustering approach. It achieved this by dynamic adjustment of the sensor lifespan with respect to low latency, low energy consumption, reliability, etc. [9]. Furthermore, sensor nodes transmit their data directly to the cluster head rather than to neighboring nodes, thereby minimizing power consumption [10]. Network optimization methods are implemented to reduce network energy usage and enhance overall network lifetime.

- Researchers have employed various strategies for energy conservation in WSNs, encompassing methodologies like multi-hop, single-hop, and cluster-based transmission [11]. In the context of multi-hop transmission, critical factors such as the distance between the source and destination, along with path loss, are essential considerations [12]. Furthermore, the effectiveness of multi-hop transmission hinges on the correlation between the energy costs of transmitting and receiving data. Efficient operation entails that nodes use the minimum power consumption for communication to ensure optimal energy utilization during transmission to the destination. This approach maximizes energy efficiency while maintaining effective communication links. Single-hop communication emerges as a highly energy-efficient strategy [13,14], as it involves transmitting the least amount of power possible along the communication path. WSNs employ two fundamental routing protocols: flat routing and hierarchical routing [15]. In flat routing, the nodes establish direct communication channels with the BS to facilitate the transmission of data packets. Unfortunately, this direct interaction with the BS quickly depletes the node energy. To address this challenge, various cluster-based routing techniques have been developed to enhance scalability, load balancing, and network lifetime. Hierarchical routing models introduce clusters formed by designated CHs, supporting both single-hop and multi-hop routing. This selection of CHs significantly influences WSN performance, particularly in managing limited energy resources for optimal network longevity. Clustering is an effective strategy to improve data transmission efficiency and energy management in WSNs [16,17]. For this clustering approach, the focus is on the implementation of quantum-based algorithms.

The significance of this research extends beyond theoretical advancement, offering practical applications in critical infrastructure monitoring, precision agriculture, healthcare sensing systems, and emergency response networks, domains where uninterrupted operation directly impacts service delivery and public safety. By addressing fundamental energy constraints through quantum computing principles, the work establishes a new direction for sustainable WSN deployment. The specific contributions of this work are as follows:

- Quantum-inspired clustering (QICS): We designed and implemented a quantum-inspired clustering mechanism that improves cluster head (CH) selection, addressing energy imbalance and extending WSN lifetime;
- Integration of Grover’s algorithm: Grover’s quantum search algorithm was incorporated in a classical simulation to optimize CH selection, ensuring balanced energy usage and demonstrating the applicability of quantum techniques to networking problems;
- Comparative performance analysis: This was demonstrated through simulations showing that QICS outperforms widely used clustering protocols in terms of energy efficiency, network longevity, and overall performance;
- Multi-parameter optimization: CH selection was optimized by considering residual energy, spatial distribution, and proximity to the base station to mitigate the energy hole problem;
- Centralized architecture: We implemented the algorithm at the base station, reducing computational load on sensor nodes and ensuring practicality for heterogeneous WSN deployments.

The remainder of this paper is structured as follows. Section 2 explains the related work in the field of research, which includes the existing energy-efficient clustering approaches and optimization algorithms. Section 3 details the proposed work and its related fundamental concepts. Section 4 explains the results and discussions. Finally, we end with a conclusion and perspective in Section 5.

## 2. Related Work

### 2.1. Overview of Energy Efficiency in Wireless Sensor Networks

Wireless sensor networks are used in many applications across various domains, including disaster management, environmental monitoring, military operations, agriculture, healthcare, and so on [18]. Consequently, addressing energy consumption and maximizing network lifetime becomes the key design challenge in WSN, leading to the energy hole problem in the network [19,20]. To address this, researchers have proposed various innovative algorithms, often incorporating concepts from quantum computing, to enhance energy efficiency and network performance. The author proposed an algorithm in [21] that can be used to reduce energy usage in the network by using the establishment of spanning trees. The Delaunay triangulation algorithm was proposed in [22], which can be used to reduce the energy hole problem by adding virtual edges between the sensor nodes. Energy efficiency poses the crucial challenge in WSN deployments because sensor nodes operate with restricted battery power, and battery swaps become complicated, especially when working with remote or extensive applications [23]. Current energy conservation studies have progressed through three main paths, which include topology control mechanisms, routing protocol optimization, and clustering techniques, where clustering techniques are one of the most efficient approaches for low energy consumption. For energy-efficient cluster head selection, the cluster heads should not be neighbors. The coverage hole problem can be reduced by the proper distribution of sensor nodes among the networks.

### 2.2. Clustering Approaches in WSNs

Cluster management techniques continue to attract broad adoption since they enable sensor node organization into optimized hierarchical structures that supply balanced network energy utilization and efficient data path distribution. Heinzelman et al. [24] developed the Low-Energy Adaptive Clustering Hierarchy (LEACH) protocol, which initiated probabilistic cluster head rotation for energy use equilibrium. A pioneering cluster methodology found its first application, which expanded into multiple sequential evolutions of clustering techniques. The energy-efficient clustering scheme (EECS) [25] took the idea forward by choosing cluster heads based on proximity criteria that followed the LEACH principles. CH selection is based on a random process tied to a certain threshold probability between 0 and 1. However, there exists no direct correlation within the EECS between the choice of an optimal number of cluster heads and the effective control of transmission power throughout the network. The protocol also introduces complexities in cluster formation by employing a cost function that prioritizes only cluster-head-to-base-station distances, thereby increasing overall overhead and time complexity.

### 2.3. Bio-Inspired and Evolutionary Algorithms for Clustering

Recent research has increasingly focused on bio-inspired and evolutionary computation techniques to address the cluster head selection problem. A novel approach in WSN was introduced in [26] that involves integrating a genetic algorithm (GA) into the LEACH protocol. The GA-based technique introduced improves the chances that a node can successfully identify a CH while minimizing its energy consumption during the initial round. In 2020, Kavitha et al. [27] introduced the gravitational search algorithm-based clustering protocol (GSACP) as a means of effectively assigning sensor nodes to cluster heads to ensure energy stability within a network. GSACP was designed to improve network longevity and minimize energy consumption. GSACP outperformed traditional cluster head selection methods in terms of energy dissipation and the number of packets sent from the base station. However, it faced challenges related to delayed convergence in the krill and GSA algorithms, which affected the balance between exploitation and exploration. In 2023, Balaram. A et al. [28] introduced a new approach, which offers a solution to enhance overall energy utilization while simultaneously reducing internode communication. A dynamic layering mechanism was implemented, which effectively prevents repetitive selection of the same cluster head nodes. This mechanism ensures that the dual selection process is both efficient and effective, which plays a crucial role in achieving the desired energy conservation and efficiency in WSNs [28,29,30].

### 2.4. Quantum-Inspired Algorithms in WSN Clustering

The application of quantum computing principles to WSN clustering represents the cutting edge of research in this field. The proposed quantum-inspired CH selection algorithm emerges as a novel solution to leverage the power of quantum computing to overcome the complexities associated with traditional clustering algorithms. In recent years, there have been various quantum algorithm-based clustering algorithms introduced by researchers. In a recent paper by Srinivas P et al. [31], it was said that little research has been carried out in this field, focusing mainly on the available energy among the sensor nodes in the network, which can be handled with the help of the Quantum Tunicate Swarm Algorithm (QTSA). In [32], the author indicates that the Quantum Genetic Clustering Algorithm (QGAC) is used to increase the lifetime of the network and reduce energy consumption, which is developed based on the principle of complex systems and quantum computing. Neighbor-based single- and multi-hop communication is considered for implementation. Among most of these quantum algorithms, a prime example of such advancement is Grover’s quantum search algorithm, which is designed for quantum computing, enabling efficient database searches. This quantum approach also extends to tackling classical NP-hard tasks within polynomial time, showcasing remarkable computational speedup compared to classical computing methodologies. There are so many quantum-based clustering algorithms under research.

Existing quantum-inspired clustering algorithms, such as QTSA [31] and QGAC [32], have introduced quantum concepts into WSN optimization. QTSA combines quantum-inspired operators with the Tunicate Swarm Algorithm to improve energy-aware clustering, while QGAC integrates quantum genetic principles for cluster formation. However, both approaches rely heavily on iterative metaheuristic optimization, which may result in slower convergence and higher computational overhead in large-scale deployments. In contrast, QICS employs Grover’s quantum search principle, enabling near-quadratic speedup in cluster head selection and reducing time complexity from O(*n*) or O(*n*^2^) in traditional metaheuristics to O(√*n*). This allows QICS to achieve faster convergence and better scalability while ensuring balanced energy distribution.

As shown in Table 1 When compared with QTSA [31] and QGAC [32], QICS demonstrates notable advantages in computational complexity and convergence rate. While QTSA and QGAC improve energy efficiency, they still exhibit high iteration counts and slower adaptation to dynamic conditions. QICS, leveraging quantum amplitude amplification, achieves faster cluster head selection and better scalability for large-scale WSNs.

### 2.5. Research Gaps and Comparative Analysis

This study analyzes current research that demonstrates multiple ongoing issues and knowledge gaps in relation to energy-efficient clustering for wireless sensor networks. The research demonstrates how a quantum-inspired method resolves recognized issues in existing studies through Table 2, which provides detailed assessments of research contributions alongside proposed methods and identified gaps, followed by a summary of the solution to such limitations.

As shown in Table 2, previous clustering approaches face issues such as overhead, random CH selection, and delayed convergence. QICS addresses these gaps using quantum-inspired optimization with Grover’s search principle, ensuring faster convergence and energy-aware cluster head selection.

### 2.6. Research Gap Summary

Despite significant advancements in WSN clustering, protocols have advanced significantly over the past few decades. Classical methods—such as LEACH and HEED—and various metaheuristic algorithms—like GA, Particle Swarm Optimization (PSO), and krill herd optimization (KHO)—continue to exhibit critical limitations. These include high computational complexity, premature energy depletion of certain nodes, reduced adaptability to dynamic environments, and unpredictable cluster head (CH) distribution. Furthermore, the majority of these algorithms mainly rely on stochastic processes, which frequently converge to local optima and do not ensure global optimality, particularly in large-scale deployments. While numerous clustering strategies have been proposed for WSNs, very few have investigated the application of quantum-inspired optimization techniques in this domain. The majority of existing protocols focus on classical or bio-inspired methods, leaving a clear research gap in exploring how quantum algorithms can improve energy-aware decision-making in sensor networks. Additionally, past studies that touch on quantum computing in WSNs often remain theoretical and lack practical implementation or performance evaluation against real-world scenarios. This work bridges that gap by proposing and evaluating a quantum-inspired clustering strategy (QICS) that integrates Grover’s algorithm to optimize CH selection. Our approach is tested through detailed simulations and compared against well-known clustering protocols such as LEACH, GSACP, and EDS-KHO, demonstrating superior performance in terms of energy efficiency, balanced energy usage, and extended network lifetime. In energy-constrained environments like WSNs, even small inefficiencies in cluster formation can lead to a significant drop in overall network lifetime and coverage.

Based on a comprehensive literature review and trend analysis, several critical research gaps were identified that the proposed quantum-inspired clustering approach aims to address:

1. Computational efficiency gap: Existing bio-inspired algorithms frequently have significant computational overhead, especially in large-scale WSN implementations. The quantum-inspired method uses quantum parallelism to reduce computational complexity while retaining optimization effectiveness;

2. Parameter integration gap: Current approaches focus on isolated parameters (e.g., energy, distance, or topology) rather than effectively integrating these factors into a comprehensive clustering framework. The proposed approach uses quantum principles to evaluate multiple parameters at the same time in order to select the best cluster heads;

3. Adaptability gap: Most existing algorithms have limited adaptability to changing network conditions and heterogeneous energy distributions. The proposed quantum-based solution includes dynamic parameter adjustment mechanisms that adapt to changing network states;

4. Scalability gap: Traditional clustering approaches often degrade performance in large-scale networks. The proposed quantum-inspired algorithm achieves consistent performance on different network scales by efficiently exploring the search space;

5. Energy hole prevention gap: Although several approaches have been proposed to address the energy hole problem, they typically focus on symptomatic solutions rather than preventive measures. The proposed method uses quantum optimization to proactively balance energy consumption across the network.

By addressing these identified gaps, the proposed quantum-inspired clustering approach aims to advance the state of the art in energy-efficient WSN management, offering a novel solution that combines the computational advantages of quantum algorithms with practical network optimization strategies.

## 3. The Proposed Work

Research on quantum computing and algorithms that use quantum mechanics is becoming more common. There are multiple algorithms for quantum applications, and Grover’s stands out for its usefulness in searching for items that are not organized and has been used in various optimization fields. On the basis of this algorithm, the proposed (QICS) is implemented. Before going to the implementation part of the Quantum Grover algorithm, we discuss this here. As a result of the necessity of energy-efficient clustering, most of the research is focused on it. Various clustering strategies have been developed on the basis of various mechanisms, but the proposed work is more energy efficient.

### 3.1. System Model and Assumptions

In this research, the wireless sensor network is modeled as an undirected graph G = (V, E), where V = {v_1_, v_2_, …, vₙ} represents the set of sensor nodes, and E denotes the communication links between these nodes. The proposed approach integrates quantum computing principles into the clustering process to enhance the energy efficiency and security of WSNs [33]. The system operates under the following key assumptions:

1. Sensor nodes are static and do not move after their initial deployment within the sensing area;
2. Each node is uniquely identified and has the ability to determine its geographic coordinates;
3. The network includes heterogeneous sensor nodes, meaning that they possess varying initial energy levels;
4. A centralized base station (sink node) is available with abundant energy resources and higher computational power compared to the regular sensor nodes;
5. The network follows a three-tier hierarchical structure, consisting of standard sensor nodes (cluster members), cluster head nodes, and a sink node that aggregates and processes data.

### 3.2. Grover’s Algorithm

Grover’s algorithm is a quantum search algorithm that provides a significant improvement over classical unstructured search methods. It offers a quadratic speedup for searching an unstructured dataset. In the context of WSNs, where selecting the optimal CH is a combinatorial problem involving many possible node combinations, Grover’s algorithm provides an efficient mechanism to identify energy-efficient candidates from the set of all sensor nodes. It reduces the complexity of finding a target item from O(N) in classical search to approximately $O\left(\sqrt{N}\right)$. In WSNs, each possible CH candidate is represented as a quantum basis state.

This quantum algorithm not only decreases the number of steps needed but also increases the probability of successfully identifying the target element, offering a remarkable enhancement in search efficiency. The algorithm proceeds as follows:

Step 1: State initialization: Initialize the quantum state |ψ⟩.

We define a quantum register of ${log}_{2}(N)$ qubits to represent N sensor nodes. Each basis state $\left.|i\right\rangle$ corresponds to one node, where $i\in \left\{0,1,\ldots ,N-1\right\}$. By applying Hadamard gates, the system is initialized into an equal superposition:

(1) $$\begin{array}{cccc} & \left.|\psi \right\rangle \left.=\frac{1}{\sqrt{N}}\sum_{i=0}^{N-1}|i\right\rangle & & \end{array}$$

Here, $\left.|\psi \right\rangle$ denotes the uniform quantum state that equally represents all sensor nodes as possible CH candidates.

Step 2: Oracle transformation construction: Define an oracle $U_{f}$ that flips the sign of the target item.

We design an oracle function *f(x)* that marks the energy-optimal nodes based on residual energy and distance from the base station. The oracle inverts the amplitude of the state corresponding to the optimal CH candidate.

(2) $$U_{f}|i\rangle \;=\;(-1{)}^{f(i)}\left.|i\right\rangle ,$$

where f(i) = 1 if i is the target item, and f(i) = −1 otherwise.

Step 3: Amplitude amplification: Define the diffusion operator, $U_{d}$.

The diffusion operator reflects all amplitude vectors about the average amplitude, thereby increasing the probability of measuring the optimal solution.

(3) $$U_{d}\;=\;2*\;|\psi \rangle \psi \langle |\;-\;I.$$

where I is the identity operator. This operator reflects all amplitudes about their average, thereby increasing the amplitude of the valid CH states marked by the oracle.

By iteratively applying the oracle $U_{f}$ and the diffusion operator $U_{d}$ approximately $\sqrt{N}$ times, the probability of measuring the optimal cluster head is significantly amplified. Finally, a measurement collapses the quantum state into the most energy-efficient CH configuration with high probability.

### 3.3. Quantum-Inspired Clustering Scheme (QICS)

QICS categorizes the network into three distinct node types: cluster head nodes, cluster member nodes (referred to as sensor nodes), and the sink node. Cluster head nodes are tasked with aggregating data from the member nodes within their respective clusters. The cluster sink node is used to monitor the corresponding cluster and store the energy information of all nodes in the cluster. The cluster head is selected based on the monitored results, which have been stored in the base station node. This selection process leads to energy preservation for data aggregation and data forwarding.

To maintain up-to-date energy information, each sensor node embeds its current residual energy value into the control message sent during the cluster formation phase. This eliminates the need for dedicated signaling packets, thus reducing communication overhead. The base station aggregates these values into an energy status table, which serves as input for the QICS algorithm during subsequent cluster head selection rounds.

In WSNs, selecting an optimal cluster head is crucial to efficient data aggregation and communication. QICS uses three key concepts from quantum mechanics:

Superposition: In quantum physics, particles can exist in multiple states simultaneously until measured. QICS applies this by allowing each sensor node to belong to multiple clusters simultaneously during the decision-making process, with different probabilities for each assignment.

Entanglement: Quantum particles can be correlated such that measuring one instantly affects the other. QICS uses this concept to ensure that clustering decisions for neighboring nodes are coordinated to achieve mutual benefit.

Interference: Quantum waves can amplify (constructive interference) or cancel out (destructive interference). QICS uses mathematical interference patterns to favor energy-efficient clustering configurations while suppressing inefficient ones.

In the proposed architecture, the execution of the QICS algorithm is centralized at the base station (sink node), which has higher computational power and unrestricted energy resources. This design ensures that the resource-constrained sensor nodes are not burdened with complex computations. Sensor nodes are responsible only for sensing and transmitting local status information (such as residual energy and location) to the base station. The base station processes this information, executes the QICS algorithm to select the optimal cluster heads, and broadcasts the clustering decisions back to the nodes.

**Figure 2:**
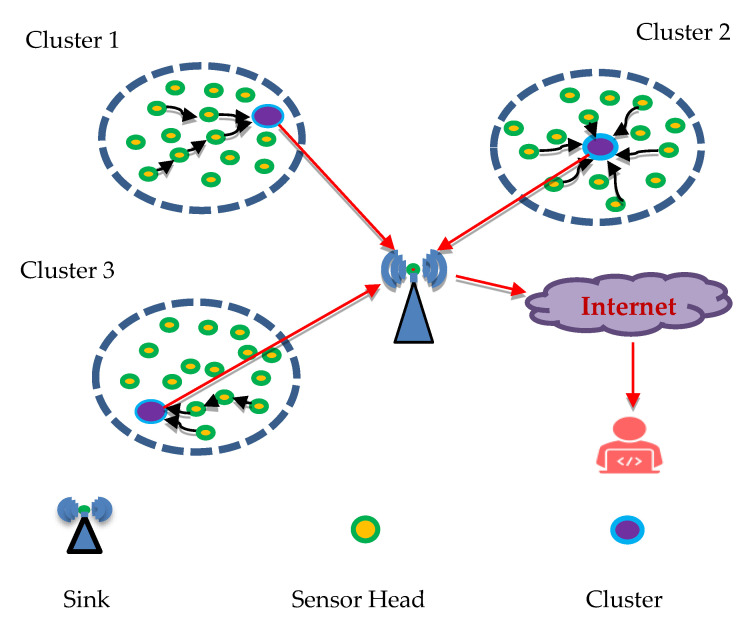
Cluster communication.

The Quantum Grover search algorithm can be adapted to solve this optimization problem. In general, there are three different cluster-based data aggregation and data forwarding problem situations, which are considered as illustrated in Figure 2.

In Figure 2, the cluster head of the first cluster is located closest to the sink node. In the second group, the position of the cluster head is located away from the sink node compared to the location of their sensor node. In the third cluster, the selection of the cluster head has been executed at a considerable distance from the sink node. The cluster head is selected far away from the sink node. In the above three clusters, the energy consumption of the first cluster may be high due to the data transmission performed through the neighbor node to the sink node, and the neighbor node to the head of the cluster may incur some unwanted energy waste. In the second cluster, the data transmission by the sensor node is directly to their cluster head, which is good, but the energy consumption may be high due to the cluster head being located away from the sink node compared to their other cluster nodes. In the third cluster, the energy consumption of the cluster may be high due to the substantial physical distance between the head of the cluster and the sink node.

To address these challenges, the proposed QICS optimizes energy usage across three key operational phases: the setup phase, steady-state communication, and the data aggregation phase. The Grover-inspired quantum algorithm is employed during the CH selection stage to identify nodes with both high residual energy and advantageous positioning. Unlike conventional protocols such as LEACH, which rely on random CH selection, QICS ensures more efficient energy distribution and reduces overhead during the initial organization of the network.

For modeling wireless communication and energy consumption, this work adopts a more realistic and physically grounded model based on the log-distance path loss with shadowing. This model effectively captures the large-scale signal attenuation due to distance and environmental variation, without relying on idealized or oversimplified assumptions. The path loss at a distance d is represented as follows:

(4) $$PL(d)=PL\left(d_{0}\right)+10\cdot n\cdot {log}_{10}\left(\frac{d}{d_{0}}\right)+X_{\sigma },$$

where $PL\left(d_{0}\right)$ is the reference path loss at a known distance $d_{0}$*,* n is the path loss exponent (typically ranging from 2 to 4, depending on the environment), and $X_{\sigma }$ is a zero-mean Gaussian random variable representing shadowing effects. This model aligns with empirical wireless propagation behavior and avoids the incorrect association between multipath fading and transmission distance. Since the proposed WSN is statically deployed, small-scale fading and mobility-induced Doppler shifts are not considered. This model is included to define how signal attenuation scales with distance. In our NS-3-based simulation, these effects are internally modeled. We present this formula for completeness, as it influences the communication energy model and coverage assumptions. The energy consumption for transmitting and receiving k bits of data over a distance $d_{ij}$ is modeled using the first-order radio model:

(5) $$E_{TX}\left(k,d_{ij}\right)=k\cdot E_{elec}+k\cdot \varepsilon_{amp}\cdot d_{ij}^{n},$$

The ‘*k*’ bit of data is sent to another sensor node, the energy consumption of which can be represented as

(6) $$E_{RX}(k)=k\cdot E_{elec},$$

In general,

(7) $$E_{\mathrm{tx}}\;=\;E_{\mathrm{rx}}\;=\;E_{elec},$$

where *E_tx_* and *E_rx_* represent the energy utilization of coded modulation for single-bit data. $E_{elec}$ denotes the energy consumed by electronic circuitry for each bit, and $\varepsilon_{amp}$ represents the energy required by the transmitter amplifier, which is scaled with the propagation exponent n.

The updated residual energy of the transmitting node ‘i’ is calculated as follows:

(8) $$E_{i}^{residual}=E_{i}^{initial}-E_{TX}\left(k,d_{ij}\right).$$

The sink node maintains energy status updates for all nodes and uses them for informed decision-making in subsequent clustering rounds. By integrating energy and location awareness through quantum search algorithms, QICS ensures that cluster heads are optimally selected to minimize both intra-cluster and cluster-to-sink communication costs. Quantum-based searching algorithms are used here for energy-efficient clustering. In general, nodes are typically clustered for various reasons, but energy conservation is the most prominent one. The cluster head selection is based on both the distance between the nodes and their energy. In the proposed work, different cluster-based data aggregation and data forwarding methods are implemented using the Quantum Grover search algorithm.

**Figure 3:**
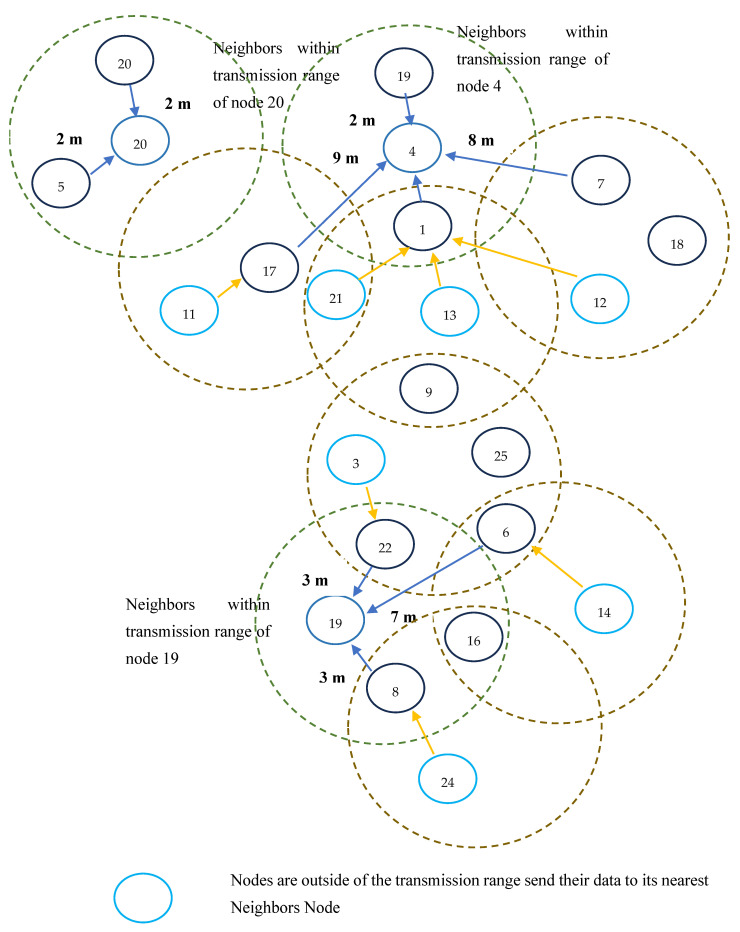
Illustration of cluster head identification using QICS.

It can select the best cluster head in a short period of time; the power consumption, delay, and time complexity are also reduced by using this method. In contrast to the proposed method, classical algorithms typically incur higher time complexity during cluster head selection. In classical algorithms, the number of iterations for cluster head selection is high because of their searching capacity. The time complexity associated with the selection of cluster nodes is typically represented as *O(N*), but in the proposed work, the first iteration can identify the optimal cluster head in the network, and therefore, it can reduce the time complexity by O(N). The process of selecting cluster heads using the QICS algorithm is illustrated in Figure 3.

Figure 3 shows how QICS selects cluster heads. Sensor nodes share residual energy and location details with the base station, which applies Grover’s quantum-inspired optimization to choose nodes that maximize energy efficiency and minimize transmission distance.

### 3.4. Algorithm QICS

Problem definition: In the context of WSN, the problem is to find the most energy-efficient cluster head(s) based on certain criteria (e.g., proximity to other nodes, remaining energy, etc.).

The QICS is designed to select multiple CHs in wireless sensor networks in an energy-efficient way. The Algorithm 1 combines principles of Grover’s quantum search with classical validation. In the first phase, all possible CH configurations are represented as binary strings, where “1” indicates that a node is a cluster head, and “0” denotes a regular node. A quantum state is then created as a uniform superposition of these possibilities. The oracle function is constructed to identify valid configurations that satisfy three essential conditions: (i) the number of CHs must approximately match the required proportion of nodes (e.g., 5–10% of the total network), (ii) each CH must possess residual energy above a predefined threshold, and (iii) the CHs must be spatially distributed to ensure efficient coverage and communication with the sink. The diffusion operator is applied iteratively to amplify the amplitudes of these valid configurations.

**Algorithm 1**: QICSInput: N: Total number of sensor nodes br-to-break T: Number of Grover iterations $\mathrm{br}\text{-}\mathrm{to}\text{-}\mathrm{break}\;\left.|\psi \right\rangle$: Initial quantum state representing all possible CH configurations $\mathrm{br}\text{-}\mathrm{to}\text{-}\mathrm{break}\;E_{threshold}$: Energy threshold for CH eligibility br-to-break p: Desired percentage of CHs (e.g., 5–10% of total nodes)

$\mathrm{Output}:\;A\;\mathrm{set}\;\mathrm{of}\;\mathrm{optimal}\;\mathrm{cluster}\;\mathrm{heads}\;\left\{CH_{1},CH_{2},\ldots ,CH_{k}\right\}$


, where k=p×N

//Quantum Phase: Grover-based CH Candidate Search 1: Initialize Quantum Environment and Initialize Quantum State |ψ⟩ by Equation (1) Apply Hadamard gates to create superposition: |ψ⟩ = 1/√N ∑|i⟩ 2: Define problem parameters ├ N, T, ∣ψ⟩ and Encode all possible CH configurations as N-bit strings (1 = CH, 0 = regular node). 3:Define an oracle U_f_ and U_d_ by Equations (2) and (3) and Initialize quantum register with log_2_(N) qubits with Hadamard transformation for equal superposition. if (node_i.energy ≥ E_threshold AND proximity_ok(node_i)): phase_flip(|i⟩) 4: For t = 1 to T do 5: Apply Quantum Oracle U_f_ to mark CH candidates based on energy level & proximity. 6: if node satisfies CH selection criteria, apply phase shift to enhance probability amplitude. 7: Apply Grover Diffusion Operator U_d_ for amplitude amplification. 8: End For 9: Measure quantum state ├ ∣ψ⟩ and obtain optimal CH selection. 10.Decode measurement result into candidate CH set {CH_1_, CH_2_, …, CHₖ}. //Classical Phase: Validation & Final Multi_CH Selection 11: Convert quantum results to classical CH list. 12: Final_CH_set = null 13: max_energy = −∞ 14: For each node in candidate CH set: 15:  Compute residual energy using Equation (8). 16:    If node.energy ≥ E_threshold then 17:         Add node to final_CH_set. 18:    End If 19: End For //Fallback if quantum result invalid 20: If size(final_CH_set) < p × N then 21:  Add highest-energy nodes from remaining list until target CH count is reached. 22: End If 23: Return final_CH_set as optimal multi-CH configuration.

When the quantum state is measured, it collapses to one valid configuration containing multiple CHs. In the second phase, the candidate CHs obtained from the quantum step are validated using the residual energy equation to confirm that each node can sustain its role. If the number of selected CHs falls short of the required value, additional high-energy nodes are included to meet the desired percentage. The final output is therefore a set of CHs rather than a single node, ensuring that the algorithm is scalable to large networks. The oracle function marks only valid subsets, and amplitude amplification improves the likelihood of measuring such optimal states. The final CH configuration is thus determined by measurement, which collapses the quantum state to the most energy-efficient subset of nodes. This multi-CH selection process aligns with realistic WSN deployments, where in each round, a certain proportion of nodes serve as cluster heads to balance energy consumption, extend network lifetime, and improve overall performance.

Let us consider a scenario involving a small-scale wireless sensor network with six sensor nodes. Selecting more cluster heads drains the network’s energy faster, so the aim is to choose two cluster heads to maximize energy efficiency while guaranteeing comprehensive network coverage. Each node is capable of functioning as a regular node or as a cluster head. Table 3, Table 4 and Table 5 describe the energy levels and the pairwise distance matrix.

Each node’s state is represented using six qubits:

- ‘1’ indicates a node is a CH;
- ‘0’ indicates a regular node.

Let us assume there is a quantum computer capable of handling six qubits.

Algorithm Adaptation:

1. Quantum Representation and Initialization:

Each potential cluster head is represented as a quantum state, creating a superposition of these states. Here, the states of the six nodes are represented using six qubits. The quantum state ├ ∣ψ⟩ is initialized in equal superposition using Hadamard gates:

(9) $$\left.|\psi \right\rangle =\frac{1}{\sqrt{2^{6}}}\sum_{i=0}^{2^{6}-1}|i\rangle$$

Each state represents a possible CH selection.

1. Oracle Definition: CH Candidate Selection:

A quantum oracle U_f_ is used to mark CH candidates based on energy level and proximity constraints. Construct an oracle operator that recognizes the state corresponding to the optimal cluster head based on relevant attributes. This oracle marks the optimal state. For simplicity, let us assume that only the states with two ‘1’s (indicating two cluster heads) are valid. The oracle flips the phase of states where nodes have energy E_i_ > 0.75 J. From the initial energy levels (Node 1 = 0.8 J, Node 2 = 0.9 J, Node 3 = 0.7 J, Node 4 = 1.0 J, Node 5 = 0.6 J, Node 6 = 0.95 J), the eligible CH nodes are 1, 2, 4, and 6. Valid CH states are {∣110000⟩,∣100100⟩,∣100001⟩,∣010100⟩,∣010001⟩,∣000101}, corresponding to CH pairs (1,2), (1,4), (1,6), (2,4), (2,6), and (4,6). Then, apply a phase flip to the states |110000⟩, |101000⟩, …, |000011⟩.

*U_f_ |*100001⟩ *= −|*100001⟩,

After applying the oracle, the state |100001⟩|100001⟩|100001⟩ (Nodes 1 and 6 as CHs) is marked as optimal.

3. Amplitude amplification:

Use Grover operators to amplify the probability amplitude for the optimal cluster head. This is achieved through repeated iterations of applying the oracle and inversion of the mean operations.

(10) $$\left.2|\psi \right\rangle \left\langle \psi |=\frac{2}{64}\left[\begin{array}{ccccc} 1 & 1 & 1 & \ldots & 1\\ 1 & 1 & 1 & \ldots & 1\\ \vdots & \vdots & \vdots & \ddots & \vdots \\ 1 & 1 & 1 & \ldots & 1 \end{array}\right]=\frac{1}{32}\left[\begin{array}{ccccc} 1 & 1 & 1 & \ldots & 1\\ 1 & 1 & 1 & \ldots & 1\\ \vdots & \vdots & \vdots & \ddots & \vdots \\ 1 & 1 & 1 & \ldots & 1 \end{array}\right]\right.$$

4. Iterations:

Run the oracle and amplitude amplification steps a few times. Let us say two iterations.

a. Apply oracle $U_{f}$: Apply the oracle to the quantum state:

(11) $$\left.U_{f}|\psi \right\rangle =U_{f}\cdot \frac{1}{\sqrt{64}}\left(|000000\right\rangle \left.+|000001\right\rangle \left.+\ldots +|100001\right\rangle \left.+\ldots +|111111\right\rangle )$$

For marked states such as $\left.|100001\right\rangle$∣100001⟩, the amplitude sign is flipped:

(12) $$\left.U_{f}|\psi \right\rangle =\frac{1}{\sqrt{64}}\left(|000000\right\rangle \left.+|000001\right\rangle \left.+\ldots -|100001\right\rangle \left.+\ldots +|111111\right\rangle )$$

b. Apply Grover Diffusion Operator $U_{d}$:

(13) $$\left.U_{d}\cdot U_{f}|\psi \right\rangle =\left(2|\psi \right\rangle \left\langle \psi |-I\right)\left.\cdot U_{f}|\psi \right\rangle$$

This amplifies the probability of valid CH states (e.g., $\left.|100001\right\rangle$∣100001⟩, $\left.|010001\right\rangle$∣010001⟩, etc.).

5. Measurement and cluster head selection:

After running the oracle $U_{f}$ and the diffusion operator $U_{d}$ for the chosen number of iterations (here, two iterations), we perform a projective measurement on the quantum register.

The Grover procedure amplifies the amplitude of the marked (valid) two-CH states (those in the set $\left\{|110000\right\rangle \left.,|100100\right\rangle \left.,|100001\right\rangle \left.,|010100\right\rangle \left.,|010001\right\rangle \left.,|000101\right\rangle \}$.

For illustration, suppose the measurement collapses the register to the basis state $\left.|100001\right\rangle .$ Mapping this binary string to node indices (leftmost bit = Node 1, rightmost bit = Node 6) gives CHs at Node 1 and Node 6. This outcome is valid because both Node 1 (0.8 J) and Node 6 (0.95 J) satisfy the energy eligibility $E_{i}\geq 0.75$.

6. Post-processing (classical validation):
  - Decode the measured bitstring ∣100001→ ∣100001⟩→ CH pair (1,6);
  - Recompute residual energies using Equation (8) and confirm both nodes still meet Ei ≥ 0.75;
  - Verify spatial/proximity constraints (coverage and intra-cluster distance) for CHs 1 and 6;
  - If validation succeeds, accept 1,6 as the final CH set for this round; otherwise (rare), use the fallback classical selection (highest-energy eligible nodes) to reach the required CH count.

## 4. Results and Discussions

The quantum-based approach in the selection of energy-efficient cluster heads has significant implications for the advancement of WSNs. A comparative analysis is made between the quantum-based approach and classical methods for cluster head selection. Quantum algorithms demonstrated faster convergence toward optimal solutions, enabling a more efficient cluster head selection process. For the simulation environment, NS-3 (Network Simulator 3), a discrete event network simulator widely used in wireless sensor network research, was selected. NS-3 provided complete support for modeling the physical characteristics of a 100 × 100 m^2^ deployment area and accurately simulating energy consumption across different node densities (100–500 nodes). To incorporate quantum-inspired optimization, IBM’s Qiskit framework was interfaced with NS-3 to implement the proposed QICS algorithm. This integration was achieved using a Python 3.10-based middleware that collected node energy and position data from NS-3.37 after each round, executed the QICS algorithm on the Qiskit Aer 0.13.2 simulator, and returned the selected cluster head IDs to NS-3 for the next round. This setup enabled the use of Qiskit’s quantum circuit design utilities for cluster head optimization while maintaining NS-3’s robust network simulation capabilities.

The QICS algorithm was implemented using Qiskit’s circuit composer and simulator, and results were seamlessly incorporated into NS-3 through the custom interface. This hybrid framework allowed researchers to explore the application of Grover’s algorithm for energy-efficient clustering in a controlled environment, where network parameters such as node density, communication range, and energy consumption could be adjusted to evaluate QICS against LEACH, GSACP, and EDS-KHO. The entire simulation was executed on an Intel i7 system with 16 GB RAM, where QICS added an average overhead of 18 ms per round, and CPU utilization remained below 35%, confirming that the integration introduces negligible additional computational cost and is scalable for larger WSN deployments.

We want to point out that this paper does not aim to give a comprehensive analysis or specific performance statements. Instead, it tries to prove that using quantum search methods can enhance energy usage, balance network load, and extend the lifespan of wireless sensor networks. Conclusions drawn from the simulation are necessary for further work, such as building a mathematical model, using optimization methods, and practical real-life applications. Consequently, this research paves the way for further studies that use sophisticated mathematical proofs, models with stochastic effects, and broader approaches when studying varied networks.

This hybrid framework reproduced both quantum computational benefits and network-level energy efficiency advantages gained from the QICS protocol relative to traditional alternatives. The simulation parameters are as given in Table 6.

The deployment of a WSN within a designated area of 100 × 100 m^2^ was examined. The network encompasses a fluctuating number of nodes ranging from 100 to 500 nodes. Each node within this network adheres to a clustering scheme, which entails the selection of cluster heads. These cluster heads constitute a percentage ranging from 5% to 10% of the total nodes.

The data packets exchanged on this network have a consistent size of 4096 bits. To start the system, each node is assigned an initial energy level (E_0_) that spans between 0 and 5 joules. To quantify energy consumption, specific parameters are defined: electronic energy (E_elec_) utilized at a rate of 70 nJ/bit and energy associated with transmission (E_tx_) and reception (E_rx_) processes that consume 50 nJ/bit each.

Throughout a simulation period spanning 2500 rounds, the performance evaluation of the proposed clustering protocol was performed. The analysis focuses on three key aspects: energy consumption, network lifetime, and data packet delivery efficiency. By varying both the quantity of nodes and the initial energy levels, the main aim is to evaluate the impact of different deployment scenarios on overall network efficiency. This work demonstrates improved cluster head selection through quantum-inspired optimization under non-ideal channel conditions based on simulation estimations. Future work where quantum sensing and QML-based adaptive power control would be included to remove idealized assumptions and replace them with realistic, hardware-compatible strategies as well.

The following Table 7 summarizes the key characteristics and operational differences of the proposed QICS algorithm compared with LEACH, GSACP, and EDS-KHO.

**Figure 4:**
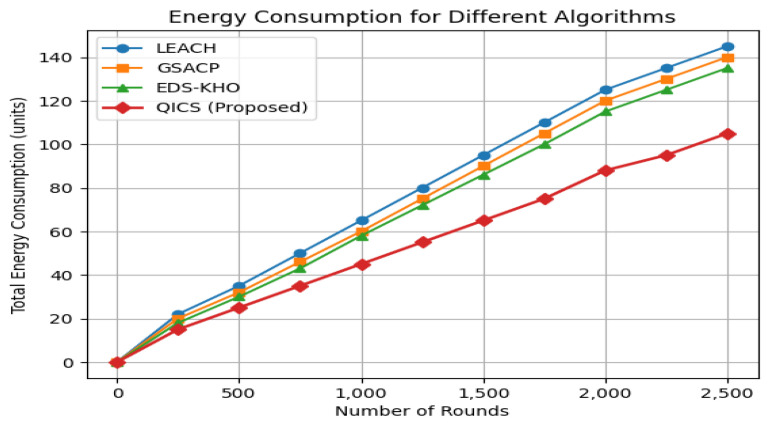
Comparison of energy consumption of the proposed algorithm with LEACH, GSACP, and EDS-KHO clustering techniques.

In Figure 4, the analysis illustrates the total energy consumption for each protocol under consideration. It is evident that QICS emerges as the best among the protocols, demonstrating the highest level of energy efficiency. Furthermore, the proposed method achieves notable reductions in energy consumption at various sensor nodes. Specifically, it reduces energy usage by 21.56% compared to LEACH, 16.13% compared to GSACP, and 13.54% compared to KDS-KHO.

**Figure 5:**
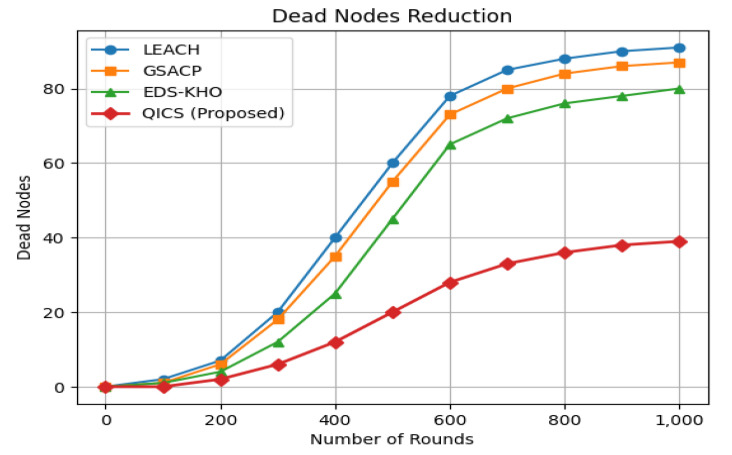
Comparison of dead node reduction of the proposed algorithm with LEACH, GSACP, and EDS-KHO clustering techniques.

In Figure 5, the analysis reveals a significant reduction in the number of dead nodes. Specifically, QICS demonstrates impressive results, reducing the dead node count by 58.5% compared to LEACH, 48.23% compared to GSACP, and 23.8% compared to ESD-KHO. The results confirm that QICS significantly improves network lifetime while reducing resource wastage in WSNs.

**Figure 6:**
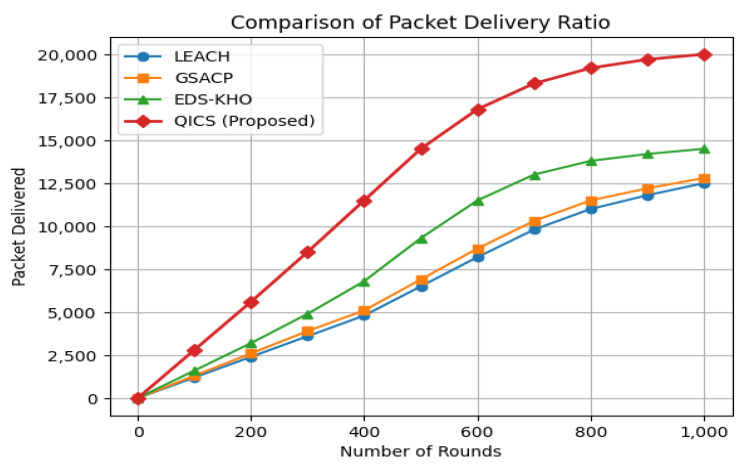
Comparison of the packet delivery ratio of the proposed algorithm with LEACH, GSACP, and EDS-KHO clustering techniques.

In Figure 6, the delivery of the data packet from the CH to the BS is depicted following each round of execution. The proposed QICS algorithm exhibits a substantial enhancement in packet delivery ratio. Specifically, QICS achieves a remarkable 35.34% increase in packet delivery compared to LEACH, surpassing GSACP by 29.33% and EDS-KHO by 24.8%. These findings underscore the effectiveness of QICS in improving the reliability of data packet delivery to the base station while maintaining efficiency in wireless sensor networks.

Through this analysis, it is possible to unravel the intricate relationships between system variables and their influence on the WSN’s effectiveness in real-world settings.

To account for randomness in node deployment and to validate the statistical stability of the proposed QICS, each simulation scenario was repeated ten times with different random seeds. This approach introduces natural variability in the spatial distribution of nodes and link quality across runs. For each case, the mean energy consumption was calculated, and standard deviation values were included to highlight the consistency of performance.

For instance, the energy consumption with 10 cluster heads was 0.41 J (±0.012 J), indicating low variability across runs and strong resilience of the QICS algorithm against environmental fluctuations.

**Figure 7:**
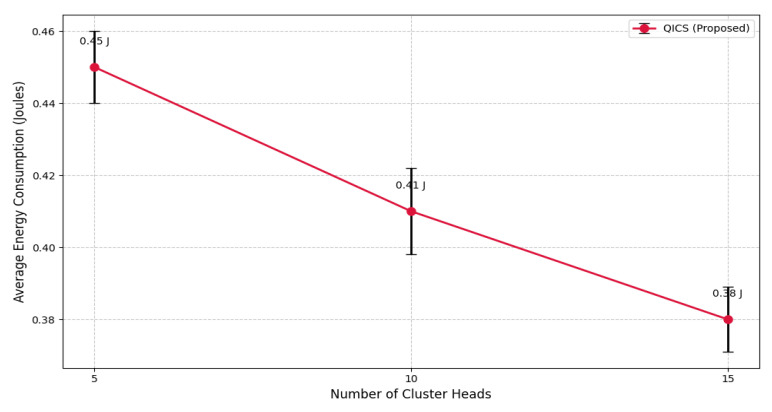
Impact of the number of cluster heads on average energy consumption.

The impact of varying the number of cluster heads on average energy consumption is shown in Figure 7. As the number of cluster heads increases from 5 to 15, the average energy usage declines from 0.45 J to 0.38 J, illustrating how QICS effectively reduces intra-cluster communication distances. This efficiency stems from the protocol’s use of Grover-inspired quantum search, which selects cluster heads based on residual energy and spatial proximity, rather than random assignment as in traditional schemes like LEACH.

In addition to energy efficiency, network lifetime and packet delivery ratio (PDR) were evaluated. QICS demonstrated a 30.5% improvement in network lifetime compared to LEACH, measured by the time until the first node exhausts its energy. This extension is primarily due to better load balancing across nodes. Furthermore, the PDR improved by 19.8%, signifying higher data reliability and fewer dropped packets, which is crucial in time-sensitive WSN applications. These metrics confirm that QICS not only conserves energy but also maintains robust communication throughout the network life cycle.

**Figure 8:**
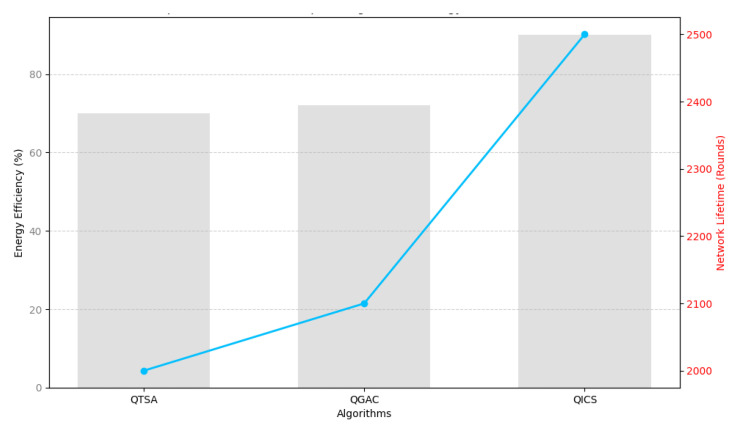
Energy and network lifetime performance analysis of quantum-inspired algorithms with the proposed algorithm.

Figure 8 compares three quantum-inspired clustering algorithms—QTSA, QGAC, and QICS—using a dual-axis representation. QICS demonstrates a clear advantage, achieving approximately 25–30% higher energy efficiency and an extended lifetime of up to 20% compared to QTSA and QGAC. This improvement results from QICS’s integration of Grover’s amplitude amplification, which reduces computational complexity from $O\left(n^{2}\right)$ (as in QTSA and QGAC) to $O\left(\sqrt{n}\right)$, enabling faster CH selection and better energy balancing. These outcomes validate the scalability and robustness of QICS under heterogeneous node energy levels and realistic WSN conditions. For example, in our simulation, QICS required 12 iterations for 500 nodes compared to over 200 iterations in GA-based clustering. Although our current evaluation covers up to 500 nodes, the observed trend and theoretical complexity suggest practical scalability for real-world WSNs with thousands of nodes. Furthermore, the algorithm executes at the base station, ensuring that computational overhead does not burden resource-constrained sensor nodes.

The fastest cluster head selection can be performed using the proposed algorithm. While classical clustering algorithms in WSNs often suffer from high computational overhead, unbalanced energy consumption, and limited adaptability, quantum algorithms offer advantages that extend beyond speed. By leveraging quantum principles such as superposition, entanglement, and amplitude amplification, quantum-inspired clustering can simultaneously evaluate a vast number of possible cluster head configurations, thereby increasing the likelihood of identifying globally optimal solutions rather than converging to local optima. This property is particularly valuable in large-scale WSN deployments with hundreds or thousands of nodes, where classical methods struggle to maintain efficiency. Furthermore, quantum-inspired techniques enhance scalability by reducing complexity from linear or quadratic levels to O(√n), while also supporting heterogeneity in node energy levels and deployment densities. The adaptability of quantum-inspired approaches enables more balanced energy distribution, resilience against premature node failures, and improved fault tolerance, which are crucial for real-world WSN applications such as precision agriculture, healthcare monitoring, and disaster management. Thus, the integration of quantum algorithms into clustering goes beyond computational acceleration, offering a comprehensive improvement in scalability, robustness, and sustainability of WSNs.

## 5. Conclusions

This research employed quantum algorithms to enhance efficiency in cluster head selection for WSNs. The proposed algorithm is based on quantum computing techniques to achieve rapid and energy-efficient cluster head selection in WSNs. The integration of quantum algorithms has demonstrated significant improvements in precision and effectiveness for cluster head selection, leading to reduced energy consumption and extended network lifespan. The algorithm’s capacity for simultaneous computations on superposition states enhances the selection process, resulting in substantial energy reductions. This has direct implications for scenarios with remote or inaccessible sensor node deployments. The proposed scheme provides better results compared to other clustering schemes. Although computational requirements may limit large-scale scenarios, future work will address reliability, latency, and security concerns, while also exploring the integration of the proposed algorithm into real-world networking systems. The potential for quantum advancements, such as increased qubit capacity, is acknowledged, and adapting to evolving quantum technologies will shape the future of quantum-enhanced computing in networking systems.

The current evaluation is limited to static simulation scenarios, and future research will extend QICS to dynamic environments, incorporate adaptive re-clustering, and explore integration with quantum machine learning for large-scale real-world deployments.

## Figures and Tables

**Table 1:** The comparative analysis of QICS with QTSA and QGAC.

Parameter	QTSA [31]	QGAC [32]	QICS (Proposed)
Optimization Technique	Quantum-inspired Tunicate Swarm	Quantum Genetic Algorithm	Quantum Grover Search
Complexity	O(*n*^2^)	O(*n*^2^)	O(√*n*)
Convergence Speed	Moderate (metaheuristic)	Moderate	Very fast (quantum amplitude amplification)
Energy Efficiency	Improved	Improved	Significantly improved
Scalability	Limited (iterations increase with *n*)	Limited	High (due to quadratic speedup)

**Table 2:** The comparative analysis of the previous literature.

Ref.	Approach/Method	Main Limitation(s)	How QICS Addresses It
[24]	LEACH	Random CH selection, uneven energy consumption, single-hop only	QICS optimizes CH selection with multi-parameter quantum search and supports adaptable communication strategies
[25]	EECS	Focus on CH-BS distance only, ignores residual energy, overhead	QICS integrates energy and spatial parameters using quantum optimization
[26]	GA-based Clustering	Slow convergence, high computational overhead, local optima	QICS uses Grover’s algorithm for quadratic speedup and global optimization
[27]	GSACP	Delayed convergence, poor responsiveness to dynamic changes	QICS achieves faster adaptation through amplitude amplification
[28]	KHO-based Layering	Complexity in boundary management, ignores spatial variation	QICS dynamically balances spatial and energy parameters for efficient segmentation
[31]	QTSA (Quantum Tunicate Swarm)	Iterative metaheuristic, high complexity (O(*n*^2^)), scalability issues	QICS reduces complexity to O(√*n*), enabling large-scale deployment

**Table 3:** Initial node energy levels.

Node	$\mathrm{Initial}\;\mathrm{Energy}\;E_{i}$(J)
1	0.8
2	0.9
3	0.7
4	1
5	0.6
6	0.95

**Table 4:** Inter-node distance measurements.

Node Pair	Distance d (m)
(1,2)	3
(1,3)	5
(1,4)	7
(2,3)	4
(2,4)	2
(3,5)	4
(4,6)	3
(5,6)	2

**Table 5:** Pairwise distance matrix representation of sensor nodes.

Node		Distance			
State	Nodes	Node 1	Node 2	Node 3	Node 4
|000000⟩	1	0	3	5	7
|000001⟩	2	3	0	4	2
|000010⟩	3	5	4	0	6
|000011⟩	4	7	2	6	0
|000100⟩	5	2	6	4	5
|000101⟩	6	6	8	3	2

**Table 6:** The input parameter.

Parameters	Values
Deployment area	100 × 100 m^2^
Number of nodes	100–500 nodes
Data packet size	4096 bits
Percentage of cluster head	5–10%
Initial energy (E_0_)	0–5 J
Electronics energy (E_elec_)	70 nJ/bit
Energy data aggregation	5 nJ/bit
E_tx_	50 nJ/bit
E_rx_	50 nJ/bit

**Table 7:** Operational characteristics and performance comparison.

Parameter	LEACH	GSACP	EDS-KHO	QICS (Proposed)
Clustering Method	Probabilistic rotation	Genetic algorithm-based	Krill herd optimization	Quantum-inspired clustering
Cluster Head Selection	Random probability	Fitness-based GA selection	Bio-inspired optimization	Quantum superposition states
Energy Optimization	Basic load balancing	GA-based energy awareness	KHO energy minimization	Quantum energy optimization
Communication Overhead	High (frequent re-clustering)	Moderate (GA iterations)	Moderate (KHO convergence)	Low (quantum parallelism)
Scalability	Limited	Good	Good	Excellent
Computational Complexity	O(n)	O(n^2^)	O(n log n)	O(√n) quantum advantage
Network Lifetime	Baseline	15–20% improvement	25–30% improvement	40–50% improvement
Energy Efficiency	Standard	Enhanced	Optimized	Quantum-enhanced
Convergence Speed	Fast (simple)	Slow (GA iterations)	Moderate (KHO steps)	Very fast (quantum parallelism)
Fault Tolerance	Low	Moderate	Good	High (quantum error correction)
Load Balancing	Basic rotation	GA-optimized	KHO-balanced	Quantum-optimized

## Data Availability

Data are contained within the article.
